# Modelling the impact of vector control interventions on *Anopheles gambiae *population dynamics

**DOI:** 10.1186/1756-3305-4-153

**Published:** 2011-07-28

**Authors:** Michael T White, Jamie T Griffin, Thomas S Churcher, Neil M Ferguson, María-Gloria Basáñez, Azra C Ghani

**Affiliations:** 1 MRC Centre for Outbreak Analysis & Modelling, Department of Infectious Disease Epidemiology, Imperial College London, London, UK

## Abstract

**Background:**

Intensive anti-malaria campaigns targeting the *Anopheles *population have demonstrated substantial reductions in adult mosquito density. Understanding the population dynamics of *Anopheles *mosquitoes throughout their whole lifecycle is important to assess the likely impact of vector control interventions alone and in combination as well as to aid the design of novel interventions.

**Methods:**

An ecological model of *Anopheles gambiae sensu lato *populations incorporating a rainfall-dependent carrying capacity and density-dependent regulation of mosquito larvae in breeding sites is developed. The model is fitted to adult mosquito catch and rainfall data from 8 villages in the Garki District of Nigeria (the 'Garki Project') using Bayesian Markov Chain Monte Carlo methods and prior estimates of parameters derived from the literature. The model is used to compare the impact of vector control interventions directed against adult mosquito stages - long-lasting insecticide treated nets (LLIN), indoor residual spraying (IRS) - and directed against aquatic mosquito stages, alone and in combination on adult mosquito density.

**Results:**

A model in which density-dependent regulation occurs in the larval stages via a linear association between larval density and larval death rates provided a good fit to seasonal adult mosquito catches. The effective mosquito reproduction number in the presence of density-dependent regulation is dependent on seasonal rainfall patterns and peaks at the start of the rainy season. In addition to killing adult mosquitoes during the extrinsic incubation period, LLINs and IRS also result in less eggs being oviposited in breeding sites leading to further reductions in adult mosquito density. Combining interventions such as the application of larvicidal or pupacidal agents that target the aquatic stages of the mosquito lifecycle with LLINs or IRS can lead to substantial reductions in adult mosquito density.

**Conclusions:**

Density-dependent regulation of anopheline larvae in breeding sites ensures robust, stable mosquito populations that can persist in the face of intensive vector control interventions. Selecting combinations of interventions that target different stages in the vector's lifecycle will result in maximum reductions in mosquito density.

## Background

Intensive anti-malaria campaigns targeting local mosquito populations with either Indoor Residual Spraying (IRS) or Long-lasting Insecticide Treated Nets (LLINs) in a number of endemic areas, particularly in Africa, have resulted in 10-50 fold reductions in the adult mosquito population and have been associated with similar declines in the prevalence of infection and incidence of disease in people living in these areas [1-4]. Understanding the determinants of such declines is crucial in the context of malaria control and elimination. Consideration of adult mosquitoes alone (the tenet of simple malaria models) is insufficient to fully understand the resulting changes in mosquito density. Instead, the entire mosquito lifecycle, including larvae, pupae and adults, and mosquito behaviour while feeding, resting and ovipositing needs to be considered. Indeed, it has recently been argued that a comprehensive understanding of vector ecology is a prerequisite for malaria elimination [5].

The gonotrophic cycle of a female *Anopheles *mosquito begins with a blood meal taken from a human or animal host. The mosquito will then rest while the blood meal is digested and the eggs in the ovaries mature. After resting and egg maturation, the mosquito is classified as gravid and begins searching for a suitable breeding site where it will lay 80-100 eggs [6,7]. The mosquito will then search for another blood meal and repeat the gonotrophic cycle. Larvae emerge from eggs and feed on bacteria, yeasts, protozoa and particulate organic matter in the water. Growth takes place via a series of moults through four morphologically distinct larval instars. Fourth instar larvae moult to become non-feeding pupae which develop into adult mosquitoes. The duration of the larval period depends mainly on temperature and, in tropical areas, lasts 7-15 days [8-10].

For a stable mosquito population to exist in a fixed environment, the population must be regulated. In ecological models this is usually assumed to be via density-dependent regulation or by a limited environmental carrying capacity. Both mechanisms lead to a limitation in the number of mosquitoes that a particular environment can sustain. In aquatic stage mosquito ecology, carrying capacity describes how many mosquito larvae/pupae an environment can support, whereas density dependence describes the intra-specific competition between larvae for food and resources, resulting in increased mortality and extended developmental times for high larval densities. The net result of increasing density-dependent competition is equivalent to decreasing the carrying capacity; however when considering interventions directed at different stages of the mosquito lifecycle, care must be taken to differentiate between these two concepts. Environmental management, e.g. filling in breeding sites, will directly reduce the carrying capacity without involving inter- or intra-specific interactions, whereas IRS and LLINs directed against adult mosquitoes will cause a reduction in oviposition and hence reduce density-dependent competition between larvae in breeding sites resulting in decreased larval mortality.

Two qualitatively different forms of density-dependent competition in the larval stages of insects have been described: contest competition where the number of larvae surviving in a breeding site reaches a limit as initial egg density increases; and scramble competition where the number of surviving larvae actually decreases at higher initial egg densities [11]. Experimental evidence suggests that density-dependent regulation of *A. gambiae *larvae follows a model of contest competition [12-14], although there are likely to be factors contributing to scramble competition, for example, cannibalism of early instar larvae by late instar larvae [15] and an increased population of predators as a result of high larval densities. Several mechanisms contribute to density-dependent regulation of mosquito larvae in breeding sites: (i) increased larval mortality at high densities; (ii) increased developmental times at higher densities; and (iii) reduced larval size, leading to smaller emerging adults at higher densities [13,14]. Although mosquito populations will be regulated by all of these factors [13,16], in this paper we focus on density-dependent larval mortality.

Vector control interventions can be targeted at several stages of the mosquito's lifecycle. Successful interventions will result in a dramatic drop in mosquito density, although interventions directed against adult mosquitoes will have the added benefit of reducing the probability that an infected mosquito survives sporogony to become infectious. Understanding mosquito population dynamics will aid in the selection of optimal packages of vector control interventions. The impact of vector control interventions on malaria transmission and adult mosquito dynamics has been widely investigated using mathematical models [17-25]. In addition a number of studies include models of the full mosquito lifecycle including eggs, larvae and adults [26-29]. In contrast to these studies we focus on the interaction between vector control interventions and the adult and aquatic stages of the anopheline vector, rather than malaria transmission between humans. Using insights from a number of entomological studies, a simple model of the *A. gambiae sensu lato *lifecycle, accounting for both the aquatic and adult stages is developed that is suitable for incorporation into existing mathematical models of malaria transmission. The model explores the comparative impact of LLINs, IRS, larvicides and pupacides, and the benefits that can be achieved by combining these interventions. The ultimate goal is to understand the combined impact of a suite of interventions in malaria endemic settings in Sub-Saharan Africa [30], with particular reference to those in which *A. gambiae *s.l. is the prevailing species.

## Methods

### Mosquito population dynamics

The aquatic part of the mosquito lifecycle consists of an egg stage, four larval instar stages and a pupal stage (the pre-imaginal stages). To reduce the complexity of the model in a biologically sensible way, the eggs and the first two larval instars are grouped into the 'early larval instar stage' *E*. The third and fourth larval instars are grouped together as 'late larval instars' *L*. The pupal stage is denoted by *P *and the female adult mosquito stage by *M*. On average each female mosquito will lay *β *eggs per day, which will hatch into early instar larvae *E*. These larvae will undergo density-dependent daily mortality at a rate *μ_E_*(*E*,*L*) which is assumed to depend on the density of both 'early' and 'late' instars. Larvae surviving the developmental period of *d_E _*days (where the reciprocal of *d_E _*is the rate of progression to the next stage) will develop into late instars *L*. These larvae will undergo density-dependent daily mortality at a rate *μ_L_*(*E*,*L*) during the *d_L _*days of development. Instars that survive development will become pupae at a rate given by the reciprocal of *d_L _*which are subjected to density independent mortality at a constant rate *μ_P _*throughout their *d_P _*days of development. It is assumed that half of all emerging adult mosquitoes are female [9]. Male adult mosquitoes are ignored, only assuming there are enough males for successful mating with females. Emerging adult female mosquitoes will search for a blood meal and begin their gonotrophic cycle. There is evidence of gonotrophic discordance in *A. gambiae *[31] whereby more than one blood meal may be required to produce a batch of eggs, but for simplicity we assume gonotrophic concordance in our model. Although there is evidence that adult mosquitoes senesce [32,33], for the sake of tractability, we make the simplifying assumption that mosquitoes undergo constant daily mortality at rate *μ_M_*. The mosquito lifecycle can thus be described by the following set of continuous ordinary differential equations,(1)

Since the model is formulated as a set of ordinary differential equations with constant rates of progression between stages, the duration of each developmental stage will be exponentially distributed. However the duration of larval and pupal development cannot be exponentially distributed as this would allow some infinitesimally short development times. This problem is overcome by stratifying the aquatic stages into the three compartments *E*, *L *and *P*, which results in an approximately gamma-shaped distribution for the developmental times from egg to pupa [34]. An alternative approach is to use the lumped age-class technique described by Hancock and Godfray [27] where a fixed time is spent in each developmental stage.

### Oviposition

A female *A. gambiae *mosquito will oviposit 80-100 eggs [6,7] per gonotrophic cycle which lasts approximately 3 days [35]. Although each individual mosquito oviposits periodically, a population of mosquitoes will lay eggs at an approximately continuous rate. The daily oviposition rate is estimated as the expected number of eggs oviposited in a mosquito's lifetime divided by the expected lifetime.(2)

If a female adult mosquito experiences constant mortality *μ_M _*throughout her lifetime and oviposits every *δ *days then the number of eggs laid per day can be estimated as(3)

Where *ε*_max _is the maximum number of eggs per oviposition, and *β*_max _is the maximum rate at which mosquitoes oviposit eggs. In practice *ε *≤ *ε*_max _and *β *≤ *β*_max _as some eggs may be oviposited in unsuitable breeding sites and some eggs may fail to hatch.

### Mosquito growth rate and reproduction number

If a female mosquito lays *ε *viable eggs per oviposition cycle, then over her lifetime she can expect to oviposit approximately  eggs where  is the proportion of mosquitoes surviving one gonotrophic cycle , is the proportion surviving two gonotrophic cycles, etc. At low larval densities when mortality rates of the stages are at their lowest, background *μ*^0 ^values, only a fraction(4)

of these eggs will survive to become imagos. The mosquito basic reproduction number *R*_0 _is defined to be the expected number of female adult mosquitoes produced per mosquito during her reproductive lifespan in the absence of regulatory constraints. In the absence of density-dependent larval death it is given by,(5)

This expression holds only in the absence of density-dependent larval mortality. In reality density-dependent effects will always be present so the actual value of *R*_eff _(the effective reproduction number) will be substantially smaller. We anticipate that more realistic estimates for *R*_eff _can be obtained using seasonal rainfall patterns and density dependence in mosquito breeding sites. Conditional on the density-regulated larval death rates *μ_E_*(*E*,*L*) and *μ_L_*(*E*,*L*) at time *t*, the mosquito reproduction number *R*_eff_(*t*) at time *t *is given by,(6)

Notice that in this expression we are assuming that the per-female mosquito fecundity and mortality rates are density independent. The value of *R*_eff_(*t*) will decrease with increasing density of early and late aquatic stages. When *R*_eff_(*t*) becomes equal to 1, each female mosquito replaces herself and the population is at a stable equilibrium.

### Density-dependent larval mortality

**Figure 1:**
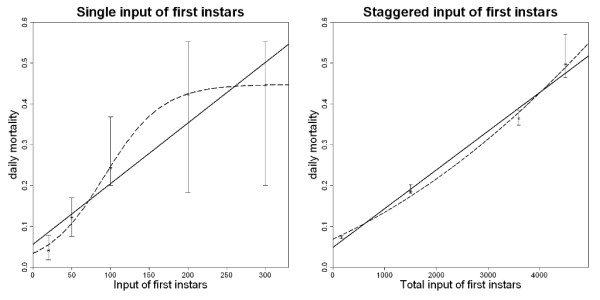
**Relationship between larval mortality rate and first instar larval density**. **A: **When batches of first instar larvae of equal age are placed in breeding sites, the daily mortality rate increases at higher larval densities. The data can be described by a linear (solid) or logistic (dashed) function. **B: **Placing batches of first instar larvae into breeding sites at staggered times results in a larval population with a mixed age structure. The data are well described by a linear (solid) or a quadratic (dashed) curve. See the Supplementary Information for further details on fitting. Error bars represent 95% confidence intervals for the data. Data are from Njunwa (1993) [12].

In a study of larval development under semi-field conditions in Tanzania by Njunwa [12], the proportion of first instar larvae introduced into artificial breeding sites that survived to emerge as adult mosquitoes was measured. Two versions of the experiment were carried out. In the first, first instar larvae were placed in the breeding site in one batch so that all larvae were the same age throughout their development. In the second, batches of first instar larvae were introduced at staggered times so that the larval population would have a varying age profile. To assess the relationship between density of instars and the daily mortality rate linear, quadratic and logistic functions were fitted to the data (Figure 1). The mortality of larvae inserted into breeding sites in one batch was well described by a logistic function, whereas the mortality of larvae inserted at staggered times was well described by a linear function although a quadratic function gave a slightly better fit (Supplementary Information). A linear relationship between density of instars and mortality was selected for the model both for the sake of parsimony and because the staggered input of larvae recreates more closely the structure of larval populations observed under natural conditions [36,37].

Mosquito aquatic stages feed and compete during the larval instar stages, and undergo morphological development but do not feed during the pupal stage. As such it is assumed that density-dependent regulation occurs at the larval instar stages only. For a breeding site containing *E*(*t*) early instar larvae and *L*(*t*) late instar larvae at time *t*, the density-dependent death rates *μ_E_*(*E*(*t*),*L*(*t*)) and *μ_L_*(*E*(*t*),*L*(*t*)) of the early and late instar larvae, respectively, at time *t *are given by,(7)

where  and  are the death rates at very low densities, *K*(*t*) is the environmental carrying capacity at time *t*, and *γ *is a factor allowing for the differential effects of density-dependent mortality on late instar larvae compared to early instar larvae. The assumption that the larval death rate is linearly proportional to the number of larvae is equivalent to the assumption made in a more complex model by Depinay *et al*. [26] where larval death rate is proportional to the total larval biomass.

### Data

Parameters for the model (see Table 1) are estimated by fitting to adult mosquito catch and meteorological data from 8 villages in the Garki District of Nigeria. The Garki Project, sponsored by the World Health Organization, was undertaken in the 1970's to investigate the feasibility of eliminating malaria from an area of intense transmission [1]. Although malaria was not eliminated during the campaign, detailed entomological, meteorological and parasitological data were collected.

**Table 1:** Prior and posterior estimates for the model parameters.

Parameter	Description	Unit	Prior median (95% CrI)	Posterior median (95% CrI)	Source of priors
*d_E_*	Development time of early larval instars	days	6.67 (4.64 - 8.69)	6.64 (4.82 - 8.53)	Bayoh & Lindsay [8]
*d_L_*	Development time of late larval instars	days	4.17 (1.88 - 6.46)	3.72 (2.03 - 5.61)	Bayoh& Lindsay [8]
*d_P_*	Development time of pupae	days	1.0 (0.20 - 1.80)	0.64 (0.07 - 1.47)	Bayoh & Lindsay [8]
	Per capita daily mortality rate of early instars (low density)	day^-1^	0.035 (0.025 - 0.044)	0.034 (0.024 - 0.044)	Bayoh & Lindsay [8]
	Per capita daily mortality rate of late instars (low density)	day^-1^	0.035 (0.025 - 0.044)	0.035 (0.025 - 0.044)	Bayoh & Lindsay [8]
*μ_P_*	Per capita daily mortality rate of pupae	day^-1^	0.25 (0.18 - 0.32)	0.25 (0.18 - 0.32)	Service [36]
*μ_M_*	Per capita daily mortality rate of adult *An. gambiae*	day^-1^	0.091 (0.082 - 0.101)	0.096 (0.087 - 0.010)	Garki project [1]
*β*	No. of eggs laid per day per mosquito	-	12.75 (0.64 - 24.86)	21.19 (11.57 - 25.31)	Service [36]
*γ*	Effect of density dependence on late instars relative to early instars	-	13.06 (9.53 - 17.36)	13.25 (9.82 - 17.51)	Service [36]
*δ*	Duration of gonotrophic cycle	days	3	-	Killeen *et al*. [35]
*ε*_max_	Maximum no. eggs per oviposition per mosquito	-	93.6	-	Hogg & Hurd [6]
*τ*	Days of rainfall contributing to carrying capacity	days	7 (3.5-11)	4 (2.5 -7)	Yé *et al*. [38]
*K*	Environmental carrying capacity	larvae	-	-	-

Notation, definition and values of the variables and parameters for the model of *A. gambiae *s.l. population dynamics.

**Figure 2:**
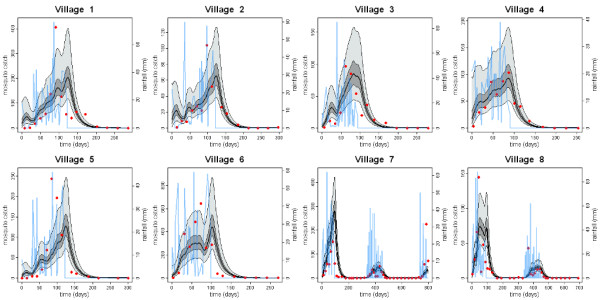
**Model fits to data from the Garki Project**. The number of adult mosquitoes trapped per night (pyrethrum spray catches of indoor resting mosquitoes) aggregated over the village is shown as red markers and compared with the model prediction. The black solid line represents the model prediction with the median posterior estimates and the envelopes depict the inter-quartile range (dark grey) and 95% credible intervals (light grey). The measured rainfall is shown in blue.

Measurements of daily rainfall were available for 8 villages. Three models for calculating the environmental carrying capacity *K*(*t*) were tested:

i) Carrying capacity proportional to the mean rainfall during the past *τ *days(8)

ii) Carrying capacity proportional to the linearly weighted mean rainfall during the past *τ *days(9)

iii) Carrying capacity proportional to past rainfall weighted by an exponential distribution with mean 2*τ*(10)

where rain(*t*) is the daily rainfall and *λ *is a fitted scaling factor unique for each village. Similar rainfall models have been used in the past to describe mosquito dynamics [38]. During extended dry periods each of these models predicts a zero carrying capacity with the consequence that no mosquito population can be sustained. This agrees with data from the Garki Project where no mosquitoes were detected during the dry season (Figure 2). However, in order for re-emergence of the mosquito population at the start of the rainy season there must be some small population of mosquitoes during the dry season [39]. When no mosquitoes are caught in pyrethrum spray catches, we assume that a mosquito could have been caught with probability 0.2. The carrying capacity during the dry season is thus set to a level capable of supporting a small mosquito population detectable 20% of the time.

The mean air temperature during the observational period of the Garki project was 24°C, fluctuating between a minimum temperature of 18°C and a maximum temperature of 30°C. Whilst daily and seasonal fluctuations in temperature are important determinants of the transmission cycle, affecting larval development times, the duration of sporogony for malaria and survival of the adult mosquito [40], temperature data in the Garki project was not consistently recorded over time and therefore we assumed a single fixed value. Larval development takes place in water, the mean temperature of which is usually higher than air temperature. In a series of experiments carried out under field conditions, Paaijmans *et al*. [41] investigated the relationship between air temperature and water temperature in typical larval breeding sites. Based on the results of these experiments, a mean air temperature of 24°C corresponds to a mean water temperature of 28°C. Priors for the parameters were based on these temperatures.

### Parameter estimation

Bayesian methods were used to fit the model to field-based adult mosquito collections (conducted to estimate indoor-resting density) and rainfall data from 8 villages in the Garki Project. Prior estimates of the model parameters are taken from the entomological literature (see Table 1). Laboratory-based studies by Bayoh and Lindsay [8] on the relationship between water temperature and *A. gambiae sensu stricto *larval and pupal developmental times and survival rates were used to provide prior estimates for the duration of each stage and the death rates at low larval densities. Several quantitative studies on the sampling of populations of *Anopheles *larvae in natural breeding sites were identified: Service [36,42], Njunwa [12] and Munga *et al*. [37] on *A. gambiae *s.l. larvae and Mwangangi *et al*. [43] on *A. arabiensis *larvae. These studies compiled sampled data into larval life tables displaying the size of the larval population stratified by instar and pupae. Data from Service [36] on larval populations sampled from marshes and borrow pits were used to obtain prior estimates of the daily oviposition rate of adult female mosquitoes and larval death rates at naturally occurring densities.

The data consisted of observations of adult mosquitoes from pyrethrum spray collections in houses in the 8 villages. Spray collections consisted of a mixture of *A. gambiae *s.s. and *A. arabiensis*. Data were aggregated to the village level for fitting and a negative binomial likelihood was chosen to capture the overdispersion in the mosquito catch data. Let *N*(*t*,*i*) denote the number of adult mosquitoes collected on day *t *in village *i*, and *M*(*t*,*i*) the number of adult mosquitoes predicted by the model. The likelihood for the parameter vector  given the data *D *can be written as(11)

where *r *is a parameter of the negative binomial distribution inversely measuring the degree of overdispersion in the mosquito catch data. Parameter estimates were obtained as the medians of the posterior distribution sampled using Markov Chain Monte Carlo (MCMC) methods. The chains were run with a burn-in of 1000 iterations (judged to have converged by visual examination of the parameter chains) which were discarded. The subsequent 1,000,000 iterations were used to estimate the posterior distribution for parameters. The best estimate of the mosquito density over time was obtained by simulating the model using the median parameter set from the posterior distribution. The 95% credible intervals around this output were obtained by repeatedly sampling parameter sets from the joint posterior distribution obtained from the final MCMC chain. Model fit was assessed visually and for different models compared using the predicted posterior probability of each model (see Supplementary Information).

### Intervention models

We consider the impact on adult mosquito density of two anti-malarial interventions directed against adult mosquitoes (LLINs and IRS) and two interventions targeting the aquatic stages (larvicide and pupacide). The following sections describe how these are incorporated into the mosquito dynamics model.

### Insecticide treated nets and indoor residual spraying

An existing model [30,44] of the effects of LLINs and IRS on the *Anopheles *vector is incorporated into our model (see Supplementary Information). We assume the vector population has the behavioural traits of the indoor and night-time biting *A. gambiae *s.s. mosquito. LLINs are assumed to have three effects on the adult mosquito population: (i) directly killing mosquitoes that land on the nets; (ii) repelling and possibly diverting mosquitoes to an animal blood host due to either insecticide irritation or the physical barrier of the net; and (iii) lengthening the duration of the gonotrophic cycle leading to a reduced oviposition rate. IRS was again assumed to have three effects on the adult mosquito population: (i) killing mosquitoes resting on walls after feeding; (ii) repelling mosquitoes from houses; and (iii) increasing the duration of the gonotrophic cycle. It was assumed that houses were sprayed with the pyrethroid lambdacyhalothrin.

### Larvicidal interventions

When a larvicide such as *Bacillus thuringiensis var. israelensis *(BTI) is applied to larval breeding sites, mosquito larvae and pupae experience increased mortality [45-47]. It is assumed that the deployment of a larvicide increases the larval and pupal background death rates ,  and *μ_P _*by a factor of *υ*, so that the number of surviving larvae is decreased. In order to mimic an 88% reduction in the number of *Anopheles *larvae as observed by Kroeger *et al*. [45], a value of *υ *= 55.2 is chosen. An 88% reduction in the number of observable larvae corresponds to a 99% reduction in the number of pupae emerging as adults, as the observed larvae are young and unlikely to survive until pupation. If a fraction *c *of breeding sites has been treated with larvicidal agents, then the mosquito population can be described by the following model,(12)

Here *E*_BTI_, *L*_BTI _and *P*_BTI _represent the early instars, late instars and pupae respectively that reside in breeding sites treated with BTI. It is assumed for tractability that larvae are distributed evenly across viable breeding sites as the purpose of this model is to obtain average behaviour for a given location which can subsequently be expanded in more detailed models. This assumption is unlikely to hold in practice as there may be a great deal of heterogeneity in breeding site preference [48-50] although the spatial scales of such heterogeneity remain poorly understood. Gu and Novak [22] have demonstrated how heterogeneity in breeding sites can be exploited if the most productive sites can be identified and targeted with larvicide, although concerns have been raised about the difficulty in identifying productive breeding sites under operational conditions [51].

### Pupacidal interventions

Pupacides preferentially target the pupal stage of the aquatic lifecycle of the *Anopheles *mosquito. Killing immature stages at the pupal stage has the additional benefit of exploiting the contribution to density-dependent regulation of those larvae that are destined to die at a later stage. The juvenile hormone analogue pyriproxyfen (PPF) kills pupae in larval breeding sites by preventing their successful development into adult mosquitoes [52,53]. In breeding sites receiving a sufficient quantity of PPF there is a 95% reduction in the number of pupae successfully emerging as adult mosquitoes (Greg Devine, personal communication). The application of PPF with efficacy *F_PPF _*to a proportion *c *of breeding sites can be described using the following model,(13)

### Parameters for Intervention Impact

To illustrate the impact of combinations of interventions the coverage parameter, *c*, is varied between 0 (zero coverage) and 1 (100% coverage). For each intervention, this coverage parameter has a slightly different interpretation. For LLINs coverage is the proportion of people sleeping under nets; for IRS coverage is the proportion of houses sprayed; for larvicides or pupacides coverage is the proportion of breeding sites that are treated. We focus on the impact of interventions after deployment before waning of insecticide efficacy becomes significant.

## Results

The model in which the carrying capacity is proportional to exponentially weighted past rainfall gave the best fit to the data (see Supplementary Information). All parameter estimates are for this model. The posterior median and 95% credible intervals (95% CrI) for the parameters are shown in Table 1. Comparisons of the predicted and observed mosquito densities for each of the 8 villages in the Garki District are shown in Figure 2.

### Mosquito growth rate and reproduction number

**Figure 3:**
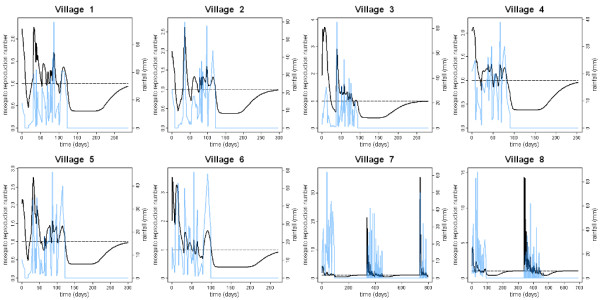
**Estimates of mosquito reproduction number**. Estimates of the effective mosquito reproduction number with density-dependent regulation at the aquatic stages for 8 villages in the Garki Project (black). The measured rainfall is shown in blue. The dashed line indicates where *R_eff _*= 1 and each female mosquito replaces herself.

In the absence of density-dependent regulation of larvae in breeding sites our parameters result in an estimate of the basic reproduction number for *A. gambiae *mosquitoes of *R_0 _*= 67 (95% CrI, 40 - 87). This value should be interpreted as a theoretical upper bound for the mosquito reproduction number as density dependence will always play some role. Estimates for the effective mosquito reproduction number *R*_eff_(*t*) for the villages in the Garki Project are shown in Figure 3. These estimates are significantly less than the upper bound of *R*_0 _= 67 as population growth is being restricted by competition during the aquatic stages. The largest peak in *R_eff _*will occur at the very start of the rainy season when existing breeding sites expand rapidly and new sites are created. For all 8 villages data collection begins just after the start of the first rainy season so the first peak in *R_eff _*is missed, although we still have *R_eff_*>1 during the growth stage of the mosquito population. For village 7 we have data for the beginning of two rainy seasons where we estimate *R_eff _*= 21.36 (95% CrI, 12.74 - 33.35) and *R_eff _*= 35.59 (95% CrI, 25.62 - 46.99). For village 8 we have data for the beginning of one rainy season where we estimate *R_eff _*= 14.38 (95% CrI, 9.29 - 24.80). For each village the value of *R_eff _*will depend on rainfall patterns and the structure of local breeding habitats. In our model the exact value of the peak in *R_eff _*will depend on the number of mosquitoes during the dry season; individual mosquitoes in small populations during the dry season will have a greater chance of reproductive success when the rains begin.

### Comparison of constant emergence and density-dependent models

**Figure 4:**
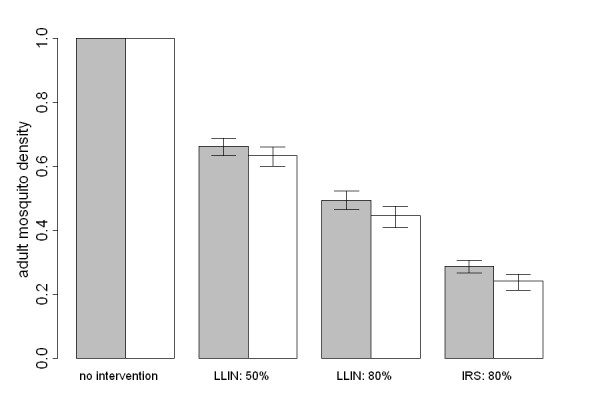
**Mosquito density as a percentage of pre-intervention density**. The density of adult mosquitoes as a percentage of total density resulting from no intervention, LLINs (at 50% and 80% coverage) and IRS at 80% coverage, predicted by a model with constant mosquito emergence (no density-dependent feedback; grey bars) in comparison with the prediction by the model including the full mosquito lifecycle presented in this paper (white bars). Error bars show 95% credible intervals arising from the uncertainty in the model fitting only. There will be additional uncertainty due to variation in the effectiveness of the interventions.

Figure 4 shows a comparison of the predicted changes in adult mosquito density due to vector control interventions for a model of constant mosquito emergence and a model of the full mosquito lifecycle with density-dependent regulation in larval breeding sites. The assumption of many vector control models that mosquitoes emerge from breeding sites at a constant rate leads to an underestimate of the reduction in adult mosquito density. This effect becomes more substantial for efficacious interventions such as IRS that are predicted to kill large numbers of adult mosquitoes and hence cause large reductions in the number of eggs oviposited in breeding sites. However the most important effect of vector control interventions on malaria transmission is still the reduction in the proportion of mosquitoes surviving the extrinsic incubation period as has been routinely highlighted in fundamental studies [54-56].

### Impact of combined vector control interventions

**Figure 5:**
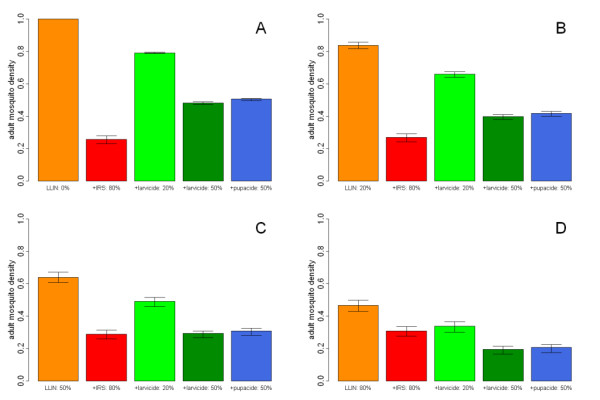
**Percentage mosquito density due to combinations of vector control interventions**. The model-predicted density of adult mosquitoes as a percentage of total density resulting from deploying IRS at 80% (red bars), larvicide at 20% (light green bars), larvicide at 50% (dark green bars), or pupacide at 50% coverage (blue bars) in combination with LLINs (orange bars) at **A**: 0% LLIN coverage, **B**: 20% LLIN coverage, **C**: 50% LLIN coverage, **D**: 80% LLIN coverage. The model corresponds to the full mosquito lifecycle. Error bars show 95% credible intervals arising from the uncertainty in the model fitting only. There will be additional uncertainty due to variation in the effectiveness of the interventions.

Vector control interventions are often deployed in settings where other interventions are already in place, most frequently LLINs [2,46,47]. Figure 5 shows the predicted changes in adult mosquito density when additional interventions are combined with LLINs at various levels of coverage. At low levels of LLIN coverage (Figures 5A and 5B) IRS causes the largest reduction in mosquito density. At higher levels of LLIN coverage (Figures 5C and 5D) larvicide and pupacide become comparable with, and in some cases, superior to IRS at reducing mosquito density. However, although larval source management may be effective at reducing mosquito numbers, it does not have the additional benefit that LLINs and IRS have in killing the adult mosquito during the malaria parasite's incubation period.

Combining interventions that target different stages of the mosquito's lifecycle can control vector populations enough to eliminate malaria transmission in some settings [5,57]. Although LLINs and IRS are highly effective interventions, they both target the adult mosquito when feeding on a human host or resting after a blood meal. Thus, if both interventions are deployed together, the barrier effect of the bed net may divert a mosquito from feeding and thus prevent the mosquito from resting on an insecticide-sprayed wall after taking a blood meal. In contrast, interventions targeting different stages of the mosquito's lifecycle, such as LLINs and larvicide, will not interfere with each other.

The larvicide BTI kills both larvae and pupae in breeding sites, and the pupacide PPF prevents pupae from emerging as adult mosquitoes. BTI and PPF are predicted to cause comparable reductions in mosquito numbers (Figure 5). The effect of increased mortality to all aquatic stages due to BTI is similar to the combined effect of PPF-induced pupal mortality and the exploitation of the contribution to density-dependent mortality of larvae destined to die as they become pupae. It may therefore fall to other factors such as cost, ease of application or impact on the ecosystem to choose between these two approaches.

## Discussion

The primary ecological factor controlling *A. gambiae *s.l. populations is considered to be density-dependent regulation of larval survival in breeding sites [13,26]. In contrast, density dependence is unlikely to be a significant factor for the adult stages of *A. gambiae *s.l. [58]. Our analysis of data from an experimental study in Tanzania suggests an approximately linear relationship between larval density and larval death rates when first instars are introduced in a staggered manner. Using this relationship in a model of mosquito dynamics, and assuming seasonal patterns in the larval carrying capacity are driven primarily by rainfall, it was possible to obtain a good fit to data on adult mosquito dynamics in 8 different villages from the Garki Project in Nigeria. Thus density-dependent regulation of the larval stages via an impact on the larval death rates provides a plausible mechanism for seasonal variation in the adult mosquito population.

The model presented here was developed for a single homogeneous site. In practice, a region's larval breeding habitats comprise multiple discrete sites as opposed to a single well mixing site [48,49,59,60]. This variation in type of breeding site, combined with the heterogeneity in breeding site preference by ovipositing females, leads to variation in the degree of density-dependent regulation, with intense intraspecific larval competition in densely populated sites and little or no competition in sparsely populated sites. This heterogeneity leads to more severe regulation of the mosquito population than a scenario where larvae are evenly distributed across all viable breeding sites. Future work will utilise the framework presented here to develop more explicit spatial models that account for this important source of heterogeneity. A second potential limitation to our model of density-dependent regulation is that eggs are oviposited in batches. This intrinsic clumping may cause competitive processes to be present even at low densities. These limitations hinder the model's ability to make predictions at mosquito densities low enough for temporary elimination of the vector population.

Although increased larval mortality is likely to be the main consequence of high larval densities, extended larval development time and the production of smaller larvae are also important factors [13,16]. Extended larval development times may delay the peak of adult mosquitoes accompanying the start of the rainy season. Smaller larvae at high densities lead to smaller emerging adult mosquitoes, which can affect the epidemiology of malaria in a number of ways: smaller mosquitoes may need to take two blood meals before their first oviposition [13]; oviposit less eggs [61]; exhibit increased mortality [62]; and may ingest a lower number of malaria parasites as they take a smaller blood meal which is digested more quickly (but see Sinden *et al*. [63] for density-dependent processes affecting *Plasmodium *sporogony itself).

Mosquito populations are sensitively dependent to climatic factors, in particular water and air temperatures and rainfall. *A. gambiae *larval survival and developmental times are dependent on water temperature [8,9,14] with faster development and decreased survival generally being observed at higher temperatures. There is only limited data on the interaction between temperature and larval density [14]. This relationship is likely to be complex and non-linear, but a model of the dynamics of larval development at a fixed temperature as demonstrated here may aid in the understanding of the dynamics at variable temperatures. Seasonal patterns of mosquito abundance closely follow seasonal patterns of rainfall. Here we assumed that the environmental carrying capacity is directly proportional to rainfall, however this may not be the case in general as it has been observed that too much rain can flush mosquito larvae from breeding sites [64]. The exact relationship between rainfall and carrying capacity is likely to be complex and dependent on local hydrological conditions.

By fitting our model to the adult mosquito data in the Garki study, we were able to obtain estimates of the variation in the adult mosquito reproduction number over time. Whilst the theoretical maximum in the absence of density-dependent regulation appears high (*R*_0 _= 67), in reality density dependence will mean that this is rarely, if ever, achieved. For the seasonal rainfall patterns in Figure 3 the effective reproduction number occurring at the beginning of the rainy season will depend on rainfall patterns and can peak at up to *R_eff _*= 35.59. However, this declines rapidly as density-dependent regulation acts and thus at any point during the malaria transmission season the value is likely to be well below this. These estimates have implications for the design of strategies that aim to reduce the adult mosquito population by reducing population growth, including the range of techniques for genetic modification being developed for *Anopheles *and other mosquito species [65,66]. For example the results shown in Figure 3 suggest that the optimal time to introduce genetically modified mosquitoes with the aim of replacement of the wild-type species is at the beginning of the rainy season when introduced mosquitoes can exploit low densities in breeding sites to reproduce efficiently.

A substantial intervention programme based on widespread LLINs and IRS can, in theory, increase adult mosquito mortality so that very few mosquitoes survive the duration of sporogony to become infectious. Eliminating the *A. gambiae *s.l. vector using interventions directed against the adult stages would require increasing adult mosquito mortality to such an extent that nearly all mosquitoes die before completing their first gonotrophic cycle. Such extreme and early mosquito mortalities are difficult to effect with current interventions and risk exerting strong selection pressures on the reproductive fitness of the mosquito population and the rapid development of insecticide resistance. Furthermore, an anti-malaria campaign based on interventions such as LLINs and IRS that targets adult mosquitoes will cause a large decrease in the mosquito population with an accompanying decrease in densities in larval breeding sites. The few mosquitoes that remain are likely to be bigger as they will have undergone less density-dependent larval competition [16]. These remaining mosquitoes may prove to be efficient transmitters of malaria. Thus the impact on adult mosquito populations, and also on malaria transmission, may be less substantial than predicted here.

The most widespread vector control interventions, LLINs and IRS, both target the adult mosquito when it is seeking to feed/has fed on its human host, thus LLINs and IRS are likely to interact inefficiently when deployed together. Our results show that, to improve upon an anti-malarial programme based on high levels of LLIN coverage, to reduce mosquito density interventions should target the mosquito at non-feeding stages. Thus, a modest level of larviciding or pupaciding may be preferable to extensive IRS campaigns (Figure 5D) if high levels (~80%) of coverage of LLINs have been obtained. A potential drawback of larviciding campaigns is the difficulty in maintaining high levels of coverage of larval breeding sites and the need for re-application of insecticide [67]. A possible exception to this could be auto-dissemination of the pupacide PPF from adult females to larval breeding sites [52] where PPF coverage on a modest proportion of walls could lead to a high level of coverage of breeding sites.

This model simulates the lifecycle of a single mixing *A. gambiae *s.l. population. In many locations other species of mosquitoes capable of transmitting malaria such as *A. funestus*, and other culicines may also be present, and interspecific interactions will be important. *A. gambiae *s.s. and *A. arabiensis *larvae compete when inhabiting shared breeding sites [9,68], but exhibit a preference for different sites, with *A. gambiae *s.s. preferring small temporary pools such as puddles and hoof prints, and *A. arabiensis *preferring larger, more permanent pools [60]. If both species share the same breeding sites then an intervention targeting the indoor-biting *A. gambiae *s.s. may facilitate species replacement with the outdoor-biting and more zoophagic *A. arabiensis*. However if both species breed in different sites, then a decrease in the *A. gambiae *s.s. population will not be accompanied by an increase in the *A. arabiensis *population. Instead, there will be an increase in the proportion of *A. arabiensis *caught compared to *A. gambiae *s.s. as observed by Bayoh *et al*. [3] in Nyanza Province in Kenya when increased ITN coverage caused a crash in the *A. gambiae *s.s. population but only minor reductions in the *A. arabiensis *population.

Many of the key anti-malarial interventions target the *Anopheles *vector, including LLINs, IRS, larviciding and environmental management of breeding sites. In addition to these existing technologies there are a number of promising vector control interventions in various phases of development, including novel insecticides, late-acting biopesticides, attractive toxic sugar bait and the introduction of genetically modified mosquitoes [53,65,69,70]. Mathematical models of mosquito population dynamics can provide insights into the likely impact of these interventions on the vector population, though consideration of possible interactions between the insecticides and the vector competence for *Plasmodium *would have to be included if projections about the likely impact on malaria transmission are also to be made. Indeed as multiple interventions are deployed targeting different stages of the mosquito's lifecycle, model predictions may prove useful in identifying combinations of interventions that interact synergistically.

## Conclusions

The dynamics of *Anopheles gambiae *populations are driven by climatic factors, rainfall and temperature, and by density-dependent competition in larval breeding habitats. In addition to providing protection to individuals against the bites of malaria infected mosquitoes, vector control interventions can also have a substantial effect on mosquito population dynamics; large reductions in mosquito numbers are frequently seen following the introduction of insecticide-treated nets or indoor residual spraying. Even greater reductions in mosquito numbers are possible by selecting combinations of interventions that target different stages in the mosquito's lifecycle, for example targeting the aquatic stages with larvicide in areas with high ITN coverage.

## Competing interests

The authors declare that they have no competing interests.

## Authors' contributions

MTW, ACG MGB and ACG conceived the analysis. JTG and MTW performed the model fitting and generated the model output. MTW wrote the first draft of the manuscript. All authors contributed to the final version of the manuscript.
